# Supplementary material for: Communicative competence assessment for learning: The effect of the application of a model on teachers in Spain
Source: PLoS One. 2020 May 29;15(5):e0233613. doi: 10.1371/journal.pone.0233613 (PMC7259689; doi:10.1371/journal.pone.0233613)
Supplement: S1 Text — (DOC) [file pone.0233613.s001.doc]

**INFORMATION FOR TEACHERS IN PARTICIPATING SCHOOLS**

The project "Improvement of the Linguistic Communication Competence of Early Childhood and Primary Education Students" has been approved in the 2013 call for R+D projects of the Ministry of Economy and Competitiveness (Spain). This project is promoted by the universities of Cordoba, Granada and Seville, and will be implemented between 2014 and 2017.

The project’s aim is the improvement of the competence in linguistic communication (from now on LCC) among primary education (PE) pupils, through an intervention based on the teaching staff training and the use of digital resources for teaching and assessment. The objectives are as follows:

- To diagnose the current situation in schools, with regard to the procedures and methodologies used for teaching competence in linguistic communication.
- To design a training programme for teachers of PE oriented to the teaching programming and formative assessment of the linguistic competence learning.
- To develop digital tools to carry out these teaching tasks.
- To apply the guidelines derived from the training received in the field of LCC in the classroom, using digital tools.
- To evaluate both the training programme and the digital resources designed for the teaching-learning process of linguistic competence, with special attention to their impact on the learning outcomes achieved in this field by the students of PE.

Regarding its method, the project is approached from a quasi-experimental approach in which systematic work and rigour in the results assessment are of essential value. In this sense, the project is designed as an experiment in which training and the use of digital tools constitute the independent variable, and the students performance in LCC becomes the dependent variable. This experiment is developed according to a pre-test/post-test design with a control group to minimize the effect of extraneous variables, so that for each experimental school there is a control school with similar characteristics.

A total number of 16 to 18 schools are taking part in the project, including both control and experimental schools, in the provinces of Cordoba, Granada and Seville.

The project has a collaborative nature, which favours the joint work of researchers and education professionals at the levels considered, contributing to the objective of improving educational practice through the professional development of teachers.

As a result of this project, it is expected that the tasks of teaching and assessment of competence in linguistic communication will be optimised among the participating schools, providing appropriate instruments for this purpose.

The participation of the experimental schools in this project implies:

| **Academic year 2014-2015** | - To facilitate the access of the research team to the school, allowing them to collect information about the way in which the teaching of linguistic communication competence is approached. It will take place in the second quarter of the 2014-2015 academic year, and will involve:   - a group interview with the teachers who teach the contents of the linguistic communication area, and  - the review of teacher planning and learning assessment documents handled by the school.   - To agree with the members of the research team on the main conclusions derived from the diagnosis carried out in the school. |
| --- | --- |
| **Academic year 2015-2016** | - To review the training programme and the digital tools designed to improve competence in linguistic communication, making contributions aimed at ensuring their suitability to the needs perceived in teaching practice. |
| **Academic year**  **2016-2017** | - To facilitate the research team to administer standardized tests to measure LCC to the 7, 9, and 11 year old students at the beginning and end of the school year. - To participate in the programmed training, and experimentally apply both the methodological guidelines derived from it and the digital tools designed for teaching and learning communication skills. - To facilitate the evaluation of the experience, providing the necessary information and assessing of the activities carried out. |

The school and the participating teachers will receive as compensation:

# Report on the situation of the teaching and learning of linguistic communication competence in schools.

# Report on the performance of the school's students in the LCC.

# Training for the teaching staff of the centre in the teaching and learning of the competence in linguistic communication.

# Experience in the use of digital tools for the programming and evaluation of this competence.

# Accreditation documents for the teaching staff, certifying their participation in the project.

**INFORMED CONSENT**

Teacher………………………………………………………..., belonging to school ………………………………………., after being informed about the project "***Improvement of the Linguistic Communication Competence of Early Childhood and Primary Education Students***", approved in the 2013 call for R+D projects, by the Ministry of Economy and Competitiveness, expresses his/her **INFORMED CONSENT AND COMMITMENT** to participate in the research during the 2014-2015 to 2016-2017 academic years and to facilitate its development, in accordance with what is expressed in the attached document on "Information for teachers in participating schools". Such participation will not imply any economic cost for the school, since the project has public funding for its development.

In Seville, on …………………… 2016.

Signed: _____________________
